# Anesthetic Efficacy of Articaine and Ketamine for Inferior Alveolar Nerve Block in Symptomatic Irreversible Pulpitis: A Prospective Randomized Double-Blind Study 

**DOI:** 10.22037/iej.v12i4.16224

**Published:** 2017

**Authors:** Vahid Sakhaeimanesh, Saber Khazaei, Naser Kaviani, Masoud Saatchi, Maryam Shafiei, Abbasali Khademi

**Affiliations:** a *Department of Endodontics, Dental Research Center, Isfahan University of Medical Sciences, Isfahan, Iran; *; b *Department of Oral and Maxillofacial Surgery, Dental Research Center, Isfahan University of Medical Sciences, Isfahan, Iran*

**Keywords:** Articaine, Inferior Alveolar Nerve Block, Irreversible Pulpitis, Ketamine

## Abstract

**Introduction:**

The aim of this prospective, randomized, double-blind study was to investigate the effect of articaine combined with ketamine on the success rate of inferior alveolar nerve block (IANB) in posterior mandible teeth with symptomatic irreversible pulpitis.

**Methods and Materials:**

Forty two adult patients with diagnosis of symptomatic irreversible pulpitis of a mandibular posterior tooth were selected. The patients received two cartridges of either containing 3.2 mL 4% articaine with epinephrine 1:200000 and 0.4 mL 50 mg/mL ketamine hydrochloride (A-ketamine group) or 3.2 mL 4% articaine with epinephrine 1:200000 and 0.4 mL normal saline (A-saline group) using conventional IANB injections. Access cavity preparation started 15 min after injection. Lip numbness was required for all the patients. Success was considered as no or mild pain on the basis of Heft-Parker visual analog scale recordings upon access cavity preparation or initial instrumentation. Data were analyzed by independent student *t*, Mann-Whitney and *Chi*-square tests.

**Results:**

The success rates were 55% and 42.9% for A-ketamine and A-saline group, respectively, with no significant differences between the two groups (*P*=0.437)**.**

**Conclusion:**

Adding 0.4 mL 50 mg/mL ketamine hydrochloride to the articaine local anesthetic did not increase the efficacy of IANB for posterior mandibular teeth with symptomatic irreversible pulpitis.

## Introduction

Inferior alveolar nerve block (IANB) is currently considered the most frequent local anesthesia technique for endodontic treatment of mandibular teeth [1, 2]. Clinical research has shown a high failure rate for this technique even with a correct application [3, 4]. On the other hand, achieving adequate anesthesia in teeth with symptomatic irreversible pulpitis has also been proven to be difficult [5-7]. Therefore, anesthetizing posterior mandibular teeth with irreversible pulpitis has been a serious challenge for the dentist, and research to solve the problem would be beneficial [7, 8].

Ketamine hydrochloride is a non-competitive antagonist of N-methyl-D-aspartate (NMDA) receptors which has analgesic effects in sub-anesthetic doses. The analgesic effect of ketamine is strong through both the central and peripheral nervous system paths [9]. It has been used alone and in combination with other local anesthetics such as lidocaine, in adeno-tonsillectomy and herniorrhaphy [9, 10]. Combination of a local anesthetic and subanesthetic dose of ketamine during surgical extraction of third molars provides good postoperative analgesia with less swelling and significantly less trismus [11]. 

It should be noted that providing an effective, safe and painless anesthesia in teeth with symptomatic irreversible pulpitis is critical [7, 12]. Kaviani *et al. *[13], showed that oral administration of ketamine might be beneficial for enhancing the anesthetic efficacy of IANB in mandibular teeth with symptomatic irreversible pulpitis. Based on ketamine analgesic effects, it may improve the success rate of anesthesia. However, anesthetic efficacy of adding ketamine to anesthetic solution in such teeth has not evaluated yet. Hence, the aim of this prospective, randomized, double-blind study was to investigate the effect of articaine plus ketamine on the success rate of IANB in posterior mandibular teeth with symptomatic irreversible pulpitis.

## Materials and Methods

Forty two adult patients participated in this prospective, randomized, double-blind study. They were referred to an approved dental clinic of Isfahan University of Medical Sciences for endodontic treatment. The Regional Ethics Committee affiliated to Isfahan University of Medical Sciences approved the study protocol (Grant No.: 388485). Also this study has been registered in the Iranian Registry of Clinical Trial (IRCT138905024440N1). A full description of the study was given to the patients with irreversible pulpitis, and an informed consent was obtained from each of the patients.

The participants of this study included the healthy (ASA I or ASA II) patients, aging between 18 to 50 years, who had a vital posterior mandibular tooth (premolar or molar) with pain and a prolonged response (in comparison with control group) to cold testing with Endo-Frost cold spray (Coltene Whaledent, Langenau, Germany) and diagnosed with irreversible pulpitis, had no severe periodontal disease, or any periapical pathological defect seen in the dental radiographs and no contraindications for the drugs and substances used in the study. The exclusion criteria were set in patients showing signs of pulp necrosis upon access cavity opening, patients showing any signs or symptoms of allergy to anesthetic solution, pregnancy and breast-feeding, patients with pain in more than one mandibular tooth, and those who refused to continue at any stage point. Therefore, each patient had a vital mandibular molar tooth with a clinical diagnosis of symptomatic irreversible pulpitis.

The patients marked their initial pain on a Heft-Parker Visual Analogue Scale (HP-VAS) [14]. The scale is a 170-mm marked line which is divided into 4 sections with different terms describing the level of pain. No pain, mild pain, moderate pain, and severe pain were indicated by 0 mm, 1-54 mm, 55-113 mm, and 114-170 mm divisions, respectively.

Articaine plus ketamine (A-ketamine) local anesthetic solution was prepared as follows: Under sterile conditions, 0.2 mL from a 1.8-mL cartridge of 4% articaine with 1:200000 epinephrine (Artinibsa; Inibsa, Barcelona, Spain) was drawn and replaced with 0.2 mL ketamine (Ketamine Hydrochloride 50 mg/mL, Rotexmedica, Trittau, Germany) using a glass micro liter syringe (Hamilton, Bonaduz, Switzerland). For articaine plus normal saline (A-saline) local anesthetic solution, 0.2 mL from a 1.8 mL cartridge of 4% articaine with 1:200000 epinephrine was drawn and replaced with 0.2 mL of normal saline. A trained dental assistant prepared the two local anesthetic formulations and coded them in a random manner. Patients were assigned equally into two groups of A-ketamine or A-saline. One operator administered two cartridges of either A-ketamine or A-saline local anesthetic solution using IANB technique for each patient in a double-blind trial. Therefore, none of the participants and operators was aware of the group assignment. All the injections were performed using a 27-gauge, 1.5-inch needle (CK ject; CK Dental, Kor-Kyungji-do, Korea) attached to a standard aspirating dental injection syringe. Lip numbness was set as a criterion to IANB achievement. If lip numbness was not profound 15 min after the injection, the patient was excluded from the study. In the present study, no participant was excluded from the study as a result of a lack of lip numbness. Then, the teeth were isolated with a rubber dam, and access cavities were prepared.

The patients were asked to rate the pain during the preparation of access cavity or initial file placement. If the patient felt pain, the treatment was stopped, and the patient marked their pain by using HP-VAS. The IANB injection was considered successful if patient felt no pain or mild pain (HP-VAS score≤54).

Data were statistically analyzed using SPSS software (SPSS version 16.0, SPSS, Chicago, IL, USA) software. Comparisons between A-ketamine and A-saline groups for the success of the IANB, gender, and tooth type differences were analyzed by *Chi *square test; age was analyzed by independent *t*-test and initial pain was analyzed using the Mann-Whitney test. The comparison was considered significant at *P*<0.05.

## Results

Out of 42 adult patients, 18 were women and 24 were men. The age range was 19-56 years, with mean±SD of 30±9.2 and 33±9.4 years in the A-ketamine and A-saline groups, respectively. The age, gender, and initial pain ratings for the A-ketamine and A-saline groups are presented in table 1. There were no differences in age, gender and initial pain between the two groups (*P*>0.05). Distribution of teeth for A-ketamine and A-saline groups is presented in table 2. There was no difference in tooth type between the two groups (*P*=0.68). The success rate of IANB was 55.0% for A-ketamine and 42.9% for A-saline group. There was no statistically significant difference in success rates between the two groups (*P*=0.437).

## Discussion

This study was carried out to investigate the effect of articaine plus ketamine on the success rate of IANB in posterior mandible teeth with symptomatic irreversible pulpitis. The results showed that the success rate of IANB in A-ketamine group was a little more than A-saline group. However, the difference was not statistically significant.

The participants’ age, sex, tooth types and initial pain records were not different between the two experimental groups; therefore, any possible effect from these factors was minimized. Lip numbness indicates successful IANB but does not guarantee pulpal analgesia or anesthesia in all patients [15-18]. Moreover, electric pulp test also seems to overestimate the rate of success when compared to the pain felt by the patients during treatment [17, 18] and cannot ensure pulpal analgesia either [19, 20]. This is attributed to the difference between pulpal anesthesia which is determined by the electric pulp test and pulpal analgesia which is determined through successful clinical pain control of the patient [18]. Therefore, the success of IANB was evaluated by measuring the pain level during endodontic access and initial instrumentation using HP-VAS, and further tests with an electric pulp tester were eliminated in this study.

The success rates of anesthesia in the case of pulpitis have been reported to be as low as 20% [15, 21] to as high as 65-70% [18, 22]. The wide range of results could be partly due to differences in the length of time when anesthesia was tested, means of testing pulpal anesthesia or analgesia (*e.g.* thermal stimulus, electrical stimulus, or clinical treatment), and population differences [16].

Combining anesthetic solution with some medications has been evaluated to overcome the local anesthetic failure of mandibular molars with irreversible pulpitis [23]. Evidence showed that mixing anesthetic solution with mannitol improved the anesthetic efficacy of IANB [24]. However, mixing anesthetic solution with some other medications such as tramadol [25], hyaluronidase [26], meperidine [15], diphenhydramine [27], sodium bicarbonate [28], carbonate [29] did not increase the anesthetic efficacy of IANB in patients with irreversible pulpitis. The results of this study showed that mixing the articaine anesthetic solution with ketamine could not increase the success rate of IANB in patients with irreversible pulpitis.

Ketamine interferes with sodium ion channels as a local anesthetic and shares with a binding site of commonly used local anesthetics, increasing their anesthetic effect [30].

Tverskoy *et al.* [31] showed that after herniorrhaphy, ulcers were infiltrated with a solution of 0.5% bupivacaine and 0.3% ketamine, and an increased level of anesthesia was demonstrated. Furthermore, they found that subcutaneous infiltration with 0.3% ketamine produced a local anesthetic effect. They claimed that ketamine acts *via* a peripheral mechanism and increases the anesthetic and analgesic actions of local anesthetics completely [31]. In addition, ketamine, as an NMDA receptor antagonist, can block the action potential of nerve fibers by affecting the sodium and potassium ion channels in their membranes. Moreover, its high lipid solubility and fast absorption in the surrounding tissue leads to slight sedative effects and also ketamine was administered as a local anesthetic in order to make use of its analgesic effects [32]. Accordingly, it was theorized that success of IANB is better in A-ketamine group than the A-saline group. Although the results of the present study showed that IANB success rate for the A-ketamine group was a little more than that for the A-saline group, although the difference was not statistically significant.

The insignificant effect of adding ketamine to articaine might be partly because of their different onset of action. The effects of ketamine following an intramuscular injection starts in approximately 30 min and lasts for about 15-30 min [32]. 

**Table 1:** Mean (SD) of preoperative values for the A-ketamine and A-saline groups

**Value**		**A-ketamine**	**A-saline**	***P-*** **value** *
**Age (y)**		30 (9.2)	33 (9.4)	0.473
**Gender**	**Women**	9	9	0.451
	**Men**	11	13	
**Initial Pain** ^**^		96 (30.2)	101 (30.0)	0.571

* *There were no significant differences between the two groups (P>0.05)*

**Table 2:** Distribution of teeth for A-ketamine and A-saline groups

**Tooth**	**A-ketamine**	**A-saline**
**First premolar**	3 (15%)	1 (5%)
**Second premolar**	5 (24%)	5 (24%)
**First molar**	7 (35%)	10 (48%)
**Second molar**	6 (30%)	5 (24%)

Intravenous injection of ketamine may results in the complete emergence of its systemic side effects such as fidgetiness, dizziness, and pulmonary distress [32]. However, in the present study a few patients in the A-ketamine group appeared to have signs of dizziness. The major limitation of the present study was the sample size. Multicenter, prospective, randomized, double-blind trials with more sample size are suggested to achieve more comprehensive results.

## Conclusion

Under the limited conditions of this study, adding 0.4 mL 50 mg/mL ketamine hydrochloride to the articaine local anesthetic did not improve the success of the IANB for posterior mandibular teeth in patients with symptomatic irreversible pulpitis.
